# Multidetector-Row CT Findings in Dogs with Different Primary Parathyroid Gland Diseases

**DOI:** 10.3390/vetsci9060273

**Published:** 2022-06-06

**Authors:** Alessia Cordella, Jessica Bertaccini, Marco Rondena, Andrea Zoia, Giovanna Bertolini

**Affiliations:** 1 Diagnostic and Interventional Radiology Division, San Marco Veterinary Clinic and Laboratory, 35030 Veggiano, Italy; alessia.cordella@outlook.com (A.C.); jessica.bertaccini@sanmarcovet.it (J.B.); 2 Pathology Division, San Marco Veterinary Clinic and Laboratory, 35030 Veggiano, Italy; marco.rondena@sanmarcovet.it; 3 Internal Medicine Division, San Marco Veterinary Clinic and Laboratory, 35030 Veggiano, Italy; andrea.zoia@sanmarcovet.it

**Keywords:** parathyroid, hyperparathyroidism, computed tomography

## Abstract

Primary hyperparathyroidism in dogs is a possibly life-threatening condition, characterized by the excess of parathyroid hormone (PTH) secretion, which leads to an increase in serum ionized calcium level. The utility of computed tomography (CT) in the detection and characterization of parathyroid diseases in dogs has not been assessed to date. Therefore, the aim of this study was to describe the use of multidetector-row CT (MDCT) for the diagnosis of parathyroid disease in dogs. For this descriptive, single-center study, the database of the San Marco Veterinary Clinic was searched for dogs having a suspicion of parathyroid disease who underwent contrast-enhanced MDCT in the period from 2005 to 2021. Dogs with histopathology of the affected parathyroid gland were subsequently considered for inclusion. A total of 22 parathyroid glands were included: 12 adenomas, 8 adenocarcinomas, and two glands with hyperplasia. Several CT features were evaluated, including parathyroid gland affected, lateralization, shape, size, attenuation, and contrast-enhancement. Although the overlap between the appearance of different diseases exists, contrast-enhanced CT was a useful method for the diagnosis of parathyroid disease in dogs.

## 1. Introduction

Dogs have two external parathyroid glands, located outside the capsule of the thyroid gland at its cranial pole, and two internal parathyroid glands, located within the caudal thyroid parenchyma [1]. Ectopic parathyroid tissue can be present in up to 6% of dogs and can be located in the neck and thorax, usually alongside the trachea [2]. Parathyroid glands secrete parathyroid hormone (PTH), an important regulator of calcium and phosphorus levels [1]. Synthesis and release of PTH is controlled by calcium receptors on the parathyroid gland, providing a rapid elevation in serum calcium in response to hypocalcemia [1]. Primary hyperparathyroidism (PHPT) results in an excess of PTH secretion, with a consequent increase in serum ionized calcium level [1]. The most common reported cause for primary hyperparathyroidism in dogs is a functional parathyroid adenoma, with adenocarcinoma and hyperplasia reported in a few cases [1]. 

Ultrasonography (US) is currently the most common imaging technique used for the assessment of parathyroid glands in dogs, although the visualization of normal parathyroid glands is considered challenging in some cases [3,4,5,6]. When they are seen on US examination, they appear as hypo/anechoic structures embedded within the thyroid parenchyma [7]. In the case of hyperparathyroidism, ultrasonography proved to be a useful modality in detecting parathyroid gland nodules, with a good correlation with surgical findings [7,8]. Different studies about the US appearance of parathyroid gland diseases showed different results [9,10]; in particular, while in one study the 3 mm cut-off was considered the best value for differentiating the hyperplastic from neoplastic gland [10], another study found that parathyroid masses measuring > 8 mm on US examination were suggestive of malignancy [9]. In a previous US study, the parathyroid gland adenocarcinomas were found to be heterogeneous, and no associations were identified between the histologic diagnosis and laterality, location, shape, or echogenicity of the parathyroid glands [10].

In previously published studies describing the normal computed tomographic (CT) and magnetic resonance (MR) appearance of the thyroid glands in dogs, the parathyroid glands could not be identified in any of the cases [11,12]. Recently, the normal CT appearance of the normal parathyroid glands in a group of healthy dogs has been described [13]. The overall visibility of the parathyroid glands was considered poor, they were ovoid-shaped and hypoattenuating with respect to the thyroid tissue both in the pre- and the post-contrast images [13]. 

The utility of CT in the diagnosis and characterization of parathyroid gland diseases in dogs has not been assessed to date. Therefore, the aim of this study was to describe the CT features of a group of dogs with confirmed histopathological parathyroid gland diseases.

## 2. Materials and Methods

For this single-center retrospective descriptive study, the electronic medical records in the clinical software (version 153, POA System 9.0, Copyright © 1993–2016, Venezia, Italy) of all dogs that had undergone CT examination between January 2005 and December 2021 were reviewed. Inclusion criteria were as follows: (1) hypercalcemia as a request for CT examination, (2) pre- and post-contrast whole body MDCT examination available for review, (3) definitive diagnosis for the cause of hypercalcemia, by histological confirmation, of primary parathyroid gland disease, and (4) available data on breed, age, sexual status (entire, neutered), bodyweight, and concomitant disease(s). Dogs were excluded when presenting a non-conclusive cytological or histological final diagnosis and if the hyperparathyroidism was secondary to chronic renal failure. All procedures were performed at the Diagnostic and Interventional Radiology Division of the San Marco Veterinary Clinic solely for the dog’s benefit and for standard diagnostic and monitoring purposes. Previous informed written consent was obtained from all dog owners. All the procedures performed complied with the European legislation “on the protection of animals used for scientific purposes” (Directive 2010/63/EU) and with the ethical requirement of the Italian law (Decreto Legislativo 04/03/2014, n. 26). 

The CT data were obtained with multidetector-row computed tomography (MDCT), either with a 16-MDCT scanner (Lightspeed 16, GE Medical Systems, Milan, Italy) or with a second or third-generation dual-source computed tomography (128 × 2 or 192 × 2 DSCT) (Somatom Definition Flash or Force; Siemens, Erlangen, Germany). For all studies, a pre-contrast series of the whole body was acquired. In all cases, a delayed phase of the whole body, including the neck region, was obtained after a delay between 2 and 6 min after contrast injection, depending on the machine used. Dogs were all placed in sternal recumbency, with the head first, forelimbs cranially extended, and the iodinated contrast agent (iohexol 370 mgI/mL, 2 mL/kg dosage followed by a saline flush) was each time injected in a cephalic vein at an injection rate of 2 mL/s using a dual-barrel injector system. For the 16-MDCT scanner, acquisition parameters were: helical modality, detector configuration 16 × 1.25 mm (50% overlap), pitch 0.562:1, and 0.7 s rotation times. All images were reconstructed using a standard algorithm (non-enhancing–non-smoothing reconstruction algorithm) with a 512^2^ matrix size and 50% overlap section thickness. Dose parameters were 120 kVp and 200 mAs. For the other studies, the scan parameters used were: 120 kVp, 400 mAs/rot (0.28 s), collimation 128/192 × 0.6 mm; images were reconstructed with 0.3 mm interval also using a non-enhancing–non-smoothing algorithm.

**Figure 1:**
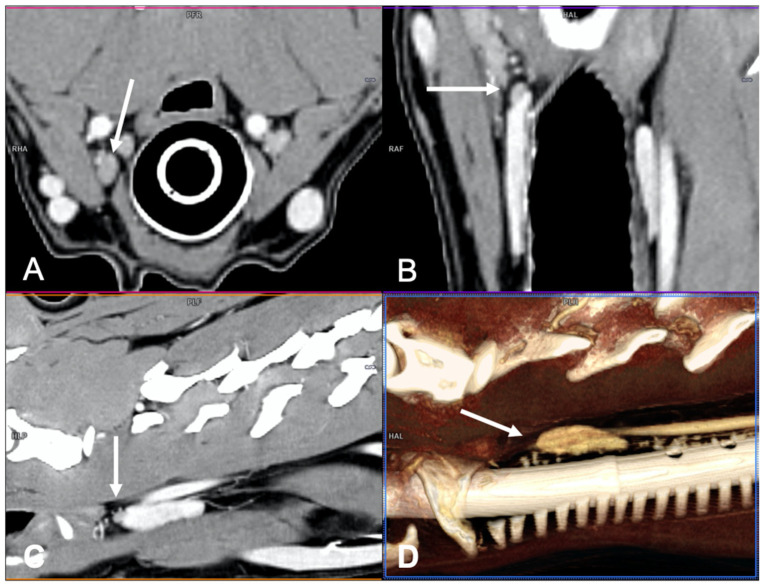
Visualization of a normal right external parathyroid gland in a dog, post-contrast images. Two different 3D image volume techniques were used: multiplanar reformation (MPR), in which the three orthogonal imaging planes are seen (**A**) transverse plane; (**B**) dorsal plane; and (**C**) sagittal plane, and volume rendering (VR) technique (parasagittal view) (**D**).

**Figure 2:**
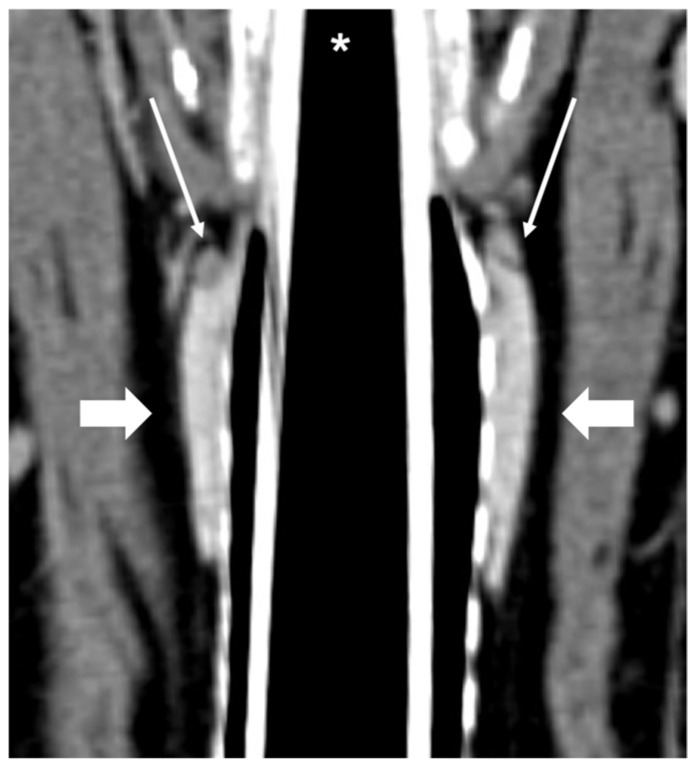
CT appearance of normal external parathyroid glands. Post-contrast dorsal CT reconstruction showing external parathyroid glands (thin arrows), visible at the cranial part of both thyroid lobes (large arrows); the left and right thyroid lobes and respective external parathyroid glands are all visible in this single image. * Trachea with endotracheal tube.

CT examinations were retrieved from the PACS (Picture Archiving Communication System—syngo.plaza, Siemens Healthineers, Milan, IT, USA) and analyzed using dedicated freestanding workstations and vendor-specific post-processing software (Syngo.Via, Siemens, Erlangen, Germany) by one author with 18 years of experience in multidetector-row CT imaging (G.B.). A combination of two-dimensional (2D) multiplanar reformations (MPRs) and three-dimensional (3D) volume rendered (VR) post-processing techniques were used (Figure 1). In particular, the dorsal reconstruction was routinely used in order to visualize both thyroid lobes and the parathyroid glands in the same image (Figure 2).

For qualitative CT analyses, the parathyroid glands were assessed for: lateralization (right/left), location (cranial/caudal), shape (rounded, ovoid, irregular), contrast enhancement (homogeneous/heterogeneous), and attenuation of the parenchyma with respect to the thyroid gland (hypoattenuating, isoattenuating, hyperattenuating). Presence of tissue and/or vascular invasion and distant metastasis was also recorded. For the quantitative assessment, the width of the parathyroid gland and the pre- and post-contrast mean attenuation (measured in Hounsfield Units—HU) were assessed. 

Due to the small sample size, statistical comparisons were made between non-malignant parathyroid glands diseases (i.e., parathyroid gland hyperplasia and parathyroid gland adenoma) versus malignant tumors (i.e., parathyroid gland adenocarcinoma). Differences among continuous variables were evaluated by non-parametric statistical analysis (Mann–Whitney test), and differences among categorical variables were compared using Fisher’s exact test or a chi-squared test. The free software R (http://www.r-project.org/, accessed on 2 June 2022) was used for statistical analyses. The level of significance was set at α = 0.05.

## 3. Results

In the selected period of time, 21 dogs fitted all the inclusion criteria, including one dog with bilateral parathyroid disease, for a total of 22 parathyroid glands included for analysis. Out of 21 dogs, 10 (48%) were crossbreed dogs, 5/21 (24%) were Dachshund, and there was 1 each of the following breeds: Beagle, Shih-Tzu, Labrador, Siberian Husky, West Highland White Terrier, and Cavalier King Charles Spaniel. A total of 13 out of 21 (62%) dogs were males (10 castrated) and 8/21 (38%) were females (7 spayed). The median age was 10 years (range: 6.5 to 15 years), and the median bodyweight was 11.2 kg (range: 3.9 to 33.7 kg).

Of the 22 abnormal parathyroid glands detected at MDCT examination, histological examination revealed parathyroid gland adenoma in 12/22 (55%) cases, parathyroid gland adenocarcinoma in 8/22 (36%) cases, and parathyroid gland hyperplasia in 2/22 (9%) cases. 

The lateralization was right-sided in 10/21 (47%) dogs and left-sided in 9/21 (43%) dogs, with one dog (5%) having bilateral parathyroid gland disease (adenoma). The external parathyroid gland (cranial) was the most commonly affected, in 18/22 (82%) cases, while the internal parathyroid gland (caudal) was affected in only 3/22 (14%) cases. One dog (5%) presented with a cranial mediastinal nodule, histologically confirmed as ectopic parathyroid adenocarcinoma. The shape of the abnormal parathyroid gland was rounded in 7/22 (32%) cases, ovoid in 11/22 (50%) cases, and irregular in 4/22 (18%) cases. The abnormal parathyroid glands were in all cases hypoattenuating compared to the adjacent thyroid tissue, both in pre-contrast and post-contrast images. The contrast enhancement of the parathyroid glands was homogeneous in 13/22 (59%) cases and heterogeneous in 9/22 (41%) cases. The presence of adjacent tissue and/or vascular invasion was not visualized in any of the patients included. Regional lymph nodes (retropharyngeal) were enlarged in 2/21 (10%) of dogs, with 1 diagnosed with adenocarcinoma and 1 with adenoma. No cytological or histological results were available for these lymph nodes. No thoracic or abdominal metastases were detected at CT examination. Median diameter of the abnormal parathyroid glands was 7 mm (range: 4 to 8.5 mm). The pre-contrast median attenuation was 37 HU (range: 6 to 62 HU) and post-contrast median attenuation was 112 HU (range: 24 to 195 HU). 

The CT findings detected in the three included parathyroid gland diseases are summarized in Table 1.

The ipsilateral parathyroid gland to the diseased parathyroid gland (external or internal) was not visible in any of the included cases. At least one contralateral parathyroid gland was detectable in CT in 12/21 (57%) cases, and the median width of these parathyroid glands was 2.7 mm (range: 2.5–3 mm). In the dog presented with ectopic parathyroid adenocarcinoma, the parathyroid glands were not visible in the expected localization (adjacent or within thyroid lobes). 

In total, 10 of the 13 dogs with a diagnosis of non-malignant parathyroid gland diseases were males (77%), while the majority of dogs (5 out of 8) with parathyroid gland carcinoma were females (63%) (*p* = 0.0708). No correlation was found between the different diseases and crossbred versus purebred status (*p* = 0.0762) or bodyweight (median 11.0 kg, range 3.9–33.7 for dogs with non-malignant parathyroid gland diseases versus median 11.8 kg, range 34.2–20.2 for dogs with parathyroid gland carcinoma; *p* = 0. 2891). 

**Figure 3:**
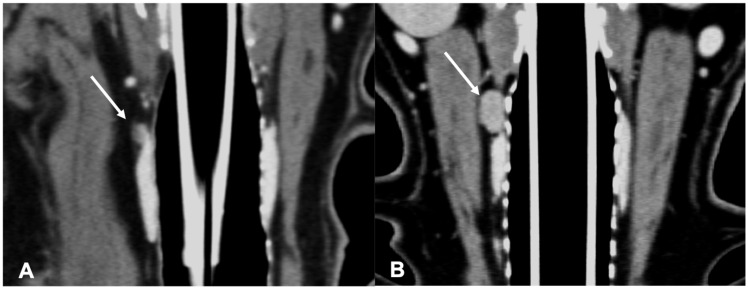
CT appearance of parathyroid gland adenoma. Post-contrast dorsal CT reconstruction in two dogs with parathyroid gland adenoma (arrow) at the level of the right external parathyroid. Note the rounded (**A**) and ovoid (**B**) shape; in both cases the parathyroid adenoma is hypoattenuating with respect to the thyroid tissue and homogenously enhancing.

The adenomas were mainly ovoid in shape (58%) and with homogeneous enhancement (67%) (Figure 3). 

**Figure 4:**
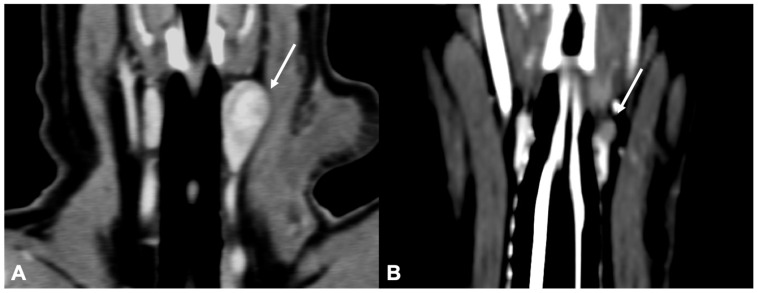
CT appearance of parathyroid gland adenocarcinoma. Post-contrast dorsal CT reconstruction in two dogs with parathyroid gland adenocarcinoma (arrow) at the level of the left external parathyroid. Note the variable CT appearance: large size of the lesion in (**A**) showing heterogeneous contrast enhancement; the adenocarcinoma in (**B**) is smaller, has a rounded shape, and homogeneous contrast enhancement.

The adenocarcinomas (Figure 4) showed variable shape and enhancement, with half of the cases showing homogeneous enhancement and half showing heterogeneous enhancement after contrast administration.

**Figure 5:**
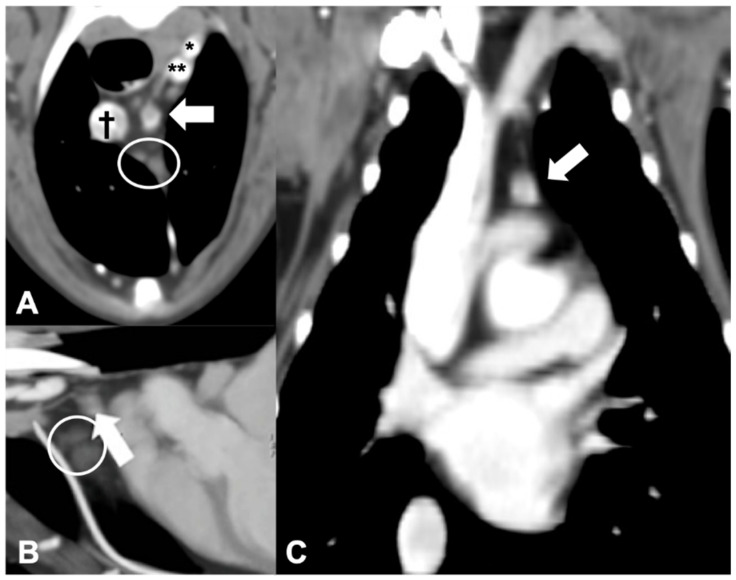
CT appearance of an ectopic parathyroid gland adenocarcinoma. Post-contrast transverse (**A**), sagittal (**B**), and dorsal (**C**) CT reconstruction images of a dog with diagnosed parathyroid gland adenocarcinoma (arrow) at the level of the cranial mediastinum. † = cranial vena cava; * = left subclavian artery; ** = brachiocephalic trunk. Note the cranial mediastinal lymph nodes (within the circle).

Only 1 of the 21 dogs included presented with primary hyperparathyroidism caused by an ectopic parathyroid adenocarcinoma. The nodule in this case was located in the cranial mediastinum, between the cranial mediastinal lymph nodes and the brachiocephalic trunk, rounded, and with heterogeneous contrast enhancement (Figure 5). Despite the localization, the presence of a single abnormal cranial mediastinal lymph node was considered unlikely and the presumptive diagnosis of ectopic parathyroid tissue was made based on the CT (than confirmed by histological examination). 

**Figure 6:**
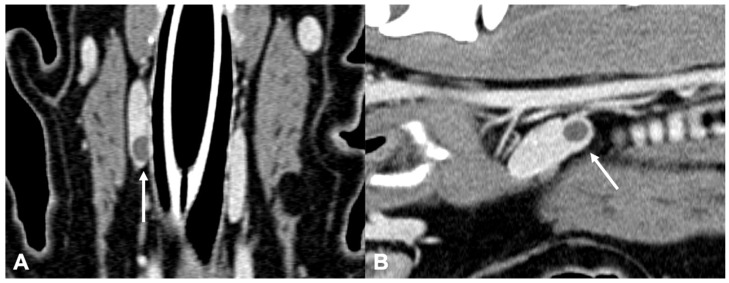
CT appearance of parathyroid gland hyperplasia. Post-contrast dorsal (**A**) and right parasagittal (**B**) CT reconstruction of one dog with parathyroid gland hyperplasia (arrow) at the level of the right internal parathyroid. The parathyroid is ovoid-shaped and homogeneously contrast-enhancing. Note the marked difference in attenuation between the parathyroid (hypoattenuating) and the adjacent thyroid tissue.

Only two dogs with parathyroid gland hyperplasia (Figure 6) were included in the study; the shape (one rounded and one ovoid) and the contrast enhancement (one homogeneous and one heterogeneous) were variable. 

In 16/21 cases, concomitant diseases were detected at CT examination and/or clinical examination: in 7 cases (33%) cystolithiasis was present, 2 cases (9%) were diagnosed with hypercortisolism, 2 (9%) with hepatic adenoma, 2 (9%) with adrenal nodules, 1 (5%) with gallbladder stones and 1 (5%) with transitional cell carcinoma of the urinary bladder. 

## 4. Discussion

The CT features of primary parathyroid diseases have not been described in veterinary medicine to date. In this study, CT examination proved to be a useful tool for the identification of parathyroid gland abnormalities and pre-surgical planning; furthermore, the CT examination is the only imaging modality that allows the identification of the lesion and the staging at the same time, particularly important in case of malignancy.

Previous studies on primary hyperparathyroidism described a breed predisposition for Keeshonds [8,14]. In our population, the majority of dogs were crossbreeds, most likely reflecting the patient demographic of the institution; among the purebred dogs, the Dachshund was overrepresented (24% of the total population included). A significant number of Dachshunds have been described in previous reports [9,10], although with a lower percentage than in our study. The median age of the study population (10 years) was in accordance to what was previously described [9,10,15]. In the current population, the median bodyweight was 11.2 kg, lower than previously reported [8,9,14]. Although in some studies, sex predilection was not assessed for parathyroid gland diseases in dogs [10,15], in our population, neutered males were overrepresented, similarly to what was previously described in one study [9]. In particular, in our population, all dogs with parathyroid gland adenoma except one were males, while a higher incidence rate of females (62%) was present in dogs affected by adenocarcinoma.

Of the 22 abnormal parathyroid glands included in this study, the majority were adenomas (55% cases), but the prevalence of parathyroid gland adenocarcinoma (36% cases) was higher than previously reported [10,15].

The external parathyroid gland (cranial) was the most commonly affected (82% cases), regardless of the type of histopathology diagnosis. Surprisingly, the majority of the parathyroid gland adenoma were right-sided, while the majority of the adenocarcinomas were left-sided. In a previous report, authors described a more common location of the adenomas and adenocarcinomas on the right cranial parathyroid gland [7], while in another report, no correlation was found between laterality or location and final diagnosis [10]. 

Computed tomographic features on shape, attenuation, size, and contrast enhancement of the three different parathyroid glands’ diseases included in this study showed some degree of overlap. In particular, the shape of the abnormal parathyroid glands was, in the majority of cases, regardless of the final diagnosis, similar to the shape of the normal parathyroid (i.e., rounded or ovoid-shaped). This result is in agreement with a previous report, in which the shape of the parathyroid gland was considered not useful to determine if the gland is normal [10]. An irregular shape of the parathyroid gland was found in our population, in some cases of adenoma or adenocarcinoma, but in no cases of hyperplasia. All the parathyroid glands included in this study showed hypoattenuating parenchyma (both pre- and post-contrast administration) with respect to the adjacent thyroid gland. This is reported to also be the CT appearance of the normal parathyroid glands in dogs [13]. Similarly, the US echogenicity of abnormal parathyroid glands was reported to be hypo to anechoic, as for the normal parathyroid glands, therefore making the echogenicity not useful to diagnose parathyroid gland disease [10]. 

The size of the normal parathyroid glands in CT was recently described to be 4.2 × 2.5 × 2.9 mm (length, width and height) [13]. In the majority of the cases included in the current study, the measured size of the abnormal parathyroid glands was larger than the reported range, as previously reported for US [6,10,15]. Previous US studies [10,16] suggested that parathyroid glands adenocarcinomas are bigger than non-neoplastic glands, typically >8 mm [9], and that a cut-off of 4 mm can be used to differentiate adenoma or carcinoma from hyperplasia [3]. No differences in size were found between the different parathyroid diseases in our population. 

The contrast enhancement of the parathyroid glands included in this study was subjectively considered either homogeneous or heterogeneous. While the majority (66%) of adenomas presented homogeneous contrast enhancement, the contrast enhancement of both adenocarcinomas and hyperplasia was variable. The pre-contrast median attenuation value of the pathological parathyroid glands included in this study was 37 HU, which was similar to the mean attenuation value of 39 HU reported for normal parathyroid glands in a previous study [13]. Additionally, the median post-contrast attenuation value (112 HU) of the pathological parathyroid glands included in this study was similar to the mean attenuation value (103 HU) of normal parathyroid glands previously reported [13]. Surprisingly, the two parathyroid gland hyperplasia included in this study showed a lower post-contrast median attenuation value than carcinoma and adenoma: 63 HU vs. 96.5 HU and 116 HU, respectively. The low number of parathyroid glands hyperplasia included in this study could have influenced this result. Nevertheless, the low post-contrast attenuation value, possibly reflecting a lower grade of vascularization, could potentially be a useful CT feature in differentiating parathyroid gland hyperplasia from adenoma and adenocarcinoma. 

No CT features of metastatic disease were detected in our population. This is in accordance with previous literature, where metastasis from parathyroid adenocarcinomas is considered rare [9,16]. Although signs of local invasiveness can be detected on histopathology [16], no evidence was found at CT examination, with all parathyroid carcinomas showing well-defined margins and marginations. Two dogs presented enlargement of the retropharyngeal lymph nodes; one was diagnosed with adenocarcinoma and one with adenoma. Unfortunately, no cytological or histological results were available for these lymph nodes (i.e., no confirmation or exclusion of metastatic disease was possible).

One of the dogs included in this study presented with hyperparathyroidism due to the presence of an ectopic parathyroid adenocarcinoma located in the cranial mediastinum. Ectopic parathyroid tissue can be detected near the thyroid lobes, adjacent to the common carotid artery cranial to the thoracic inlet, and rarely in the mediastinum [7,17]. Mediastinal ectopic parathyroid tissue could not be detected on US examination [7], making the CT examination the modality of choice for the visualization of ectopic parathyroid tissue, especially in this unusual location. 

The ipsilateral parathyroid gland (external or internal) was not visible at CT examination in any of the patients included in the study, but one contralateral parathyroid gland was detectable in 12/21 (57%) cases, with similar dimensions as previously reported for normal parathyroid glands [13]. These results could be related to the reported difficulty to detect normal parathyroid glands with cross-sectional imaging in dogs [11,12], or to the atrophy of the other parathyroid gland in case of primary hyperparathyroidism, due to hypersecretion of PTH from the pathological gland [18].

Seven of the included dogs had the concomitant presence of urolithiasis. This is in accordance to previous reports, in which an association between the hypercalcemia in dogs with primary hyperparathyroidism and the presence of uroliths was suspected [15,16].

Limitations of this study include the relative low number of cases included, and the non-uniformity of the non-malignant parathyroid group, which was represented by two different parathyroid glands diseases (i.e., parathyroid gland hyperplasia and parathyroid gland adenoma). A further limitation is represented by the fact that some of the CT features were subjectively evaluated, and due to the small size of the lesions, they could have been evaluated differently by other observers. Furthermore, the pathologic parathyroid gland could potentially mimic the presence of a thyroid nodule; further studies are needed in order to evaluate the use of CT to distinguish between the two diseases. 

In conclusion, contrast-enhanced CT was a useful method for the diagnosis and characterization of parathyroid disease in dogs. The external parathyroid gland was most commonly affected; adenomas were more frequently right-sided, while adenocarcinomas were more frequently left-sided. In many cases, the CT features of the different diseases were similar. For this reason, and due to the limited number of cases included in this case-series, further studies are necessary to assess associations between CT features and histological diagnosis of parathyroid gland diseases in dogs. 

## Figures and Tables

**Table 1:** CT features of different parathyroid diseases.

Diagnosis	Hyperplasia (2) *	Adenoma (12) *	Adenocarcinoma (8)	*p*-Value
Lateralization	Right: 1	Right: 8	Right: 2	0.1271
	Left: 1	Left: 4	Left: 5	
			Ectopic (thorax): 1	
Localization	External: 1	External: 11	External: 6	1
	Internal: 1	Internal: 1	Internal: 1	
Shape	Rounded: 1	Rounded: 3	Rounded: 3	0.6563
	Ovoid: 1	Ovoid: 7	Ovoid: 3	
	Irregular: 0	Irregular: 2	Irregular: 2	
Contrast enhancement	Homogeneous: 1	Homogeneous: 8	Homogeneous: 4	0.5119
	Heterogeneous: 1	Heterogeneous: 4	Heterogeneous: 4	
** Size (mm)	7 (6–8)	7.5 (4–12)	7 (5–14)	0.9442
** Pre-contrast attenuation (HU)	35 (34–36)	37 (6–62)	38 (28–49)	0.8181
** Post-contrast attenuation (HU)	63 (24–102)	116 (57–179)	96.5 (74–195)	0.8650

* For statistical analysis, data from hyperplasia and adenocarcinoma (i.e., non-malignant parathyroid diseases) are combined together and compared versus adenocarcinoma. ** Size and pre- and post-attenuation HU are presented as median and range; HU = Hounsfield Units.

## Data Availability

Not Applicable.
